# *Fodinicola acaciae* sp. nov., an Endophytic Actinomycete Isolated from the Roots of *Acacia mangium* Willd. and Its Genome Analysis

**DOI:** 10.3390/microorganisms8040467

**Published:** 2020-03-25

**Authors:** Huyền Thị Thanh Phạm, Wipawadee Suwannapan, Wilaiwan Koomsiri, Yuki Inahashi, Akira Také, Atsuko Matsumoto, Arinthip Thamchaipenet

**Affiliations:** 1 Department of Genetics, Faculty of Science, Kasetsart University, Chatuchak, Bangkok 10900, Thailand; phamthithanhhuyen.h@ku.th (H.T.T.P.); wipawadee.s@ku.th (W.S.); wilaiwan.ko@ku.th (W.K.); 2 Omics Center for Agriculture, Bioresources, Food and Health, Kasetsart University (OmiKU), Bangkok 10900, Thailand; 3 Kitasato Institute for Life Sciences, Kitasato University, 5-9-1 Shirokane, Minato-ku, Tokyo 108-8641, Japan; y-ina@lisci.kitasato-u.ac.jp (Y.I.); amatsu@lisci.kitasato-u.ac.jp (A.M.); 4 Department of Microbiology, Kitasato University School of Medicine, 1-5-1 Kitasato, Minami-ku, Sagamihara, Kanagawa 252-0374, Japan; a.take@med.kitasato-u.ac.jp

**Keywords:** endophytic actinomycete, new species, *Fodinicola*, genome analysis

## Abstract

A novel endophytic actinomycete strain GKU 173^T^ isolated from the roots of *Acacia mangium* Willd. showed potential plant growth promoting (PGP) activity. Phylogenetic analysis, based on 16S rRNA gene, indicated that strain GKU 173^T^ was the most closely related to *Fodinicola feengrottensis* HKI 0501^T^—the only species in the genus *Fodinicola*. Morphology and chemotaxonomy of strain GKU 173^T^ indicated that it belongs to the genus *Fodinicola* by having meso-diaminopimelic acid in the cell wall and xylose as the characteristic cell-wall sugars. The cellular fatty acid profile mainly comprised iso-C_16:0_, anteiso-C_17:0_, iso-C_18:0_, and iso-C_17:0_. The major menaquinones were MK-9(H_4_), MK-9(H_6_), and MK-9(H_8_). The main polar phospholipids contained diphosphatidylglycerol (DPG), phosphatidylethanolamine (PE), and phosphatidylinositol (PI). Genome analysis signified DNA G+C content of 67.81 mol%. The level of digital DNA-DNA relatedness between strain GKU 173^T^ and the type strain was 21.30%. On the basis of polyphasic characteristics, strain GKU 173^T^ clearly represents a novel species of the genus *Fodinicola*, for which the name *Fodinicola* *acaciae* sp. nov. (= TBRC 10620^T^ = NBRC 114213^T^) is proposed. Furthermore, genome analysis of both strains suggested that members of the genus *Fodinicola* are promising sources of beneficial PGP-actinomycetes and novel secondary metabolites.

## 1. Introduction

Endophytic actinomycetes colonize the internal plant tissues and usually have a beneficial effect to the host plant by promoting growth and protecting the plant from biotic and abiotic stresses without any damage or morphological changes to the plant [1,2]. Isolation and identification of novel genus and species of endophytic actinomycetes are so far attractive since these organisms are potential sources for plant growth-promoting (PGP) bacteria. Various PGP activity, including production of auxin to enhance plant growth, siderophores to chelate iron and other elements, 1-aminocyclopropane-1-carboxylate (ACC) deaminase to reduce plant stress ethylene, and solubilization of inorganic phosphate, were identified from several endophytic actinomycetes [3,4,5]. Reports evidencing that those endophytic actinomycetes directly improve and promote plant growth and protect plants against pathogens and abiotic stresses have been gradually increasing [5,6,7]. Furthermore, endophytic actinomycetes are recognized for their capability to produce new antimicrobial metabolites [8]. Recent genome mining of actinomycetes has revealed a remarkably large number of secondary metabolite biosynthetic gene clusters (BGC) [9], including substantial numbers of silent/cryptic BGCs that are potential sources for novel bioactive compounds which have not been detected under conventional method [10]. 

The genus *Fodinicola* was first proposed by Carlsohn et al. [11] and is a member in the family *Cryptosporangiaceae* [12] within the order *Cryptosporangiales* [13]. Member of the genus only comprises a single species, *Fodinicola feengrottensis* HKI 0501^T^, which was isolated from acidic metal-containing rocks of a medieval alum slate mine in Germany [11]. Generally, the species of this genus produce branched substrate mycelium and irregular rod-like fragmented aerial hyphae. The genus is characterized chemotaxonomically by the presence of meso-diaminopimelic acid in the cell wall, and xylose as the diagnostic cell-wall sugars. The predominant menaquinones are MK-9(H4), MK-9(H6) and MK-9(H8). The polar lipids comprise diphosphatidylglycerol, phosphatidylethanolamine, phosphatidylserine, and phosphatidylinositol. Phylogenetically, the genus is closely affiliated to the genus *Cryptosporangium* [14]. In this work, a new species of endophytic actinomycete belonging to the genus *Fodinicola* was described by polyphasic taxonomy. Furthermore, the whole genome sequences of the novel species and the single member of the genus *Fodinicola* were investigated for PGP related genes and specialized BGCs.

## 2. Material and Methods

### 2.1. Isolation and Culture Conditions 

The roots of a black wattle tree (*Acacia mangium* Willd.), collected at Kasetsart University, Bangkok, Thailand (13°50′34″ N, 100°34′30″ E), were surface-sterilized following the previously described method [15]. Colonies were appeared on starch-casein agar [16] supplemented with ketocanozol (50 µg/mL), nalidixic acid (25 µg/mL), and nystatin (50 µg/mL) after incubation at 28 °C for two weeks. A single colony was purified and cultured on mannitol-soybean (MS) agar [17] and maintained in 20% (v/v) glycerol at ‒80 °C. 

### 2.2. Characterization of Plant Growth-Promoting Traits

Indole-3-acetic acid (IAA) was determined by colorimetric method [18]. Strain GKU 173^T^ was grown in glucose-beef extract broth supplemented with 10 mM L-tryptophan at 28 °C for 7 days in dark. The culture was then centrifuged and 2 mL of supernatant was mixed with 1 mL of Salkowski’s reagent (0.5 M FeCl_3_, 70% perchloric acid). The positive reaction was indicated by development of a pink-red in color and was quantified by measuring absorbance at 530 nm compared with a standard curve of pure IAA [18]. Phosphate solubilization was determined by the development of clear zone around the bacterial plug on Pikovskaya’s agar [19] supplemented with 2% (w/v) tricalcium phosphate after incubation at 28 °C for three weeks. Siderophore production was detected by the development of orange-yellow halo around the bacterial plug on chrome azurol S (CAS) agar [20] after incubation at 28 °C for three weeks. 1-Aminocyclopropane-1-carboxylate (ACC) deaminase activity was verified by positive growth on minimal medium containing 3mM ACC as a sole nitrogen source after incubation at 28 °C for 21 days in comparison with (NH_4_)_2_SO_4_ (2 g/L) [21]. 

### 2.3. 16S rRNA Gene Sequencing and Phylogenetic Analysis

Genomic DNA of strain GKU 173^T^ was extracted from the culture grown on MS agar at 28 °C for seven days according to standard procedure [22]. 16S rRNA gene was amplified using specific primers and PCR condition, as previously described [23]. The PCR product was purified by GenepHlow^TM^ Gel/PCR kit (Geneaid, New Taipei City, Taiwan) and was sent for sequencing at Macrogen (Seoul, Republic of Korea). The almost full-length 16S rRNA gene sequences of strain GKU 173^T^ was compared with 16S rRNA gene sequences of reference type strains using the EzBioCloud server database [24]. Multiple sequence alignments were performed using ClustalW tool in MEGA version 7.0 [25] and trimmed manually where necessary. Phylogenetic evolutionary trees were constructed with the neighbor-joining [26], maximum-likelihood [27] and maximum-parsimony [28] algorithms using the MEGA software. Evolutionary distance was calculated using Kimura’s two-parameter model [29]. The topologies of the phylogenetic trees were evaluated using the bootstrap method [30] base on 1000 resampled datasets.

### 2.4. Morphological and Physiological Characteristics 

Morphological and physiological characteristics of strain GKU 173^T^ and *Fodinicola feengrottensis* HKI 0501^T^ (JCM 14718^T^) were determined under the same condition. Gram staining was carried out using standard Gram staining method. Both strains were cultivated on Bennett’s agar [31], MS agar, organic medium 79 [32], and International *Streptomyces* Project (ISP) media [33] namely, yeast extract-malt extract agar (ISP 2), oatmeal agar (ISP 3), inorganic salt-starch-agar (ISP 4), glycerol-asparagine agar (ISP 5), peptone-yeast extract-iron agar (ISP 6), and tyrosine agar (ISP 7) for up to 21 to 28 days at 28 °C. The color of mycelia and soluble pigment were determined by comparison with color chips from Methuen Handbook of Color [34]. Strain GKU 173^T^ was grown on oatmeal-nitrate agar (ON: 0.02% KNO_3_, 0.02% MgSO_4_.7H_2_O, 0.05% K_2_HPO_4_, 0.3% oatmeal, and 1.5% agar) at 28 °C for 21 days for observation of hyphae and spore morphology under scanning electron microscopy (JSM-5600, JEOL, Tokyo, Japan). Spore motility was observed under light microscope followed the method of Tamura et al. [35]. 

Growth of strain GKU 173^T^ at different temperatures (5‒50 °C) was tested on MS agar slants for 15 days using a temperature gradient incubator (model TN-3, Toyo Kagaku Sangyo, Tokyo, Japan). The pH range for growth (pH values 3‒11, at an interval of 1.0 pH unit) was performed on MS agar adjusting pH values with 1 M NaOH and 1 M HCl. Growth at different NaCl concentration [0‒7% (w/v), at an interval of 1%] was determined on ISP 3 agar for three weeks. Catalase and oxidase activities were observed with 3% (v/v) hydrogen peroxide solution and 1% (v/v) tetramethyl-p-phenylenediamine solution [36], respectively. Acid production from carbohydrates was determined based on the method of Gordon et al. [37]. The utilization of carbon and nitrogen sources were determined on ISP 9 and basal medium supplemented with a final concentration of 1% carbon and 0.1% nitrogen sources, respectively [38]. Degradation of Tweens 20 and 80 [1% (w/v)] were tested on Sierra medium [39] after incubation at 28 °C for 21 days. Decomposition of adenine and tyrosine [0.5% (w/v) each], xanthine, hypoxanthine, xylan [0.4% (w/v) each], casein [1% (w/v) skimmed milk] and urea were determined according to the previous methods [37,38]. Hydrolysis of starch was tested by flooding the culture growing on ISP 4 agar with Lugol’s solution [40]. Milk coagulation and peptonization were performed in 10% (w/v) skimmed milk broth (Difco, BD, Franklin Lakes, NJ, USA) and observed after incubation at 37 °C for 21 days. Gelatin liquefaction was examined on gelatin medium consisted of 0.5% peptone (Difco, BD, Franklin Lakes, NJ, USA), 2% glucose, 20% gelatin, pH 7. Reduction of nitrate was performed using nitrate broth (Difco, BD, Franklin Lakes, NJ, USA). Enzyme activities were tested by the API ZYM test kit (bioMérieux, Durham, NC, USA), according to the manufacturer’s instruction.

### 2.5. Chemotaxonomic Characterization 

For chemotaxonomic analysis, biomass was prepared by growing strain GKU 173^T^ in Bennett’s broth on a rotary sharker at 28 °C for 14 days. Cells were harvested by centrifugation, washed with distilled water, and freeze-dried. Cell wall peptidoglycan was prepared using the modified method described by Kawamoto et al. [41] to analyze the cell-wall amino acids and sugars. The amino acid composition of the peptidoglycan hydrolysate (6 M HCl, 100 °C, 16 h) was determined by one-dimensional TLC on a cellulose plate (Merck, Burlington, MA, USA), using solvent system n-buthanol:acetic acid:water (4:1:2, v/v) and then analysed by LC/MS following the method of Také et al. [42] after *N*^α^-(5-fluoro-2,4-dinitrophenyl)-D-leucinamide (FDLA) derivatization according to the method of Fujii et al. [43]. The LC/MS analysis was performed by AB Sciex TripleTOF 5600+ System with a CAPCELL CORE C18 (3.0 mm x 100 mm) column (Shiseido, Tokyo, Japan) using eluent A (water with 0.1% formic acid) and eluent B (methanol with 0.1% formic acid) by gradient elution: 0–2 min, 50% B; 2–18 min, 50–100% B; 18–20 min, 100% B; flow rate, and 0.5 mL/min. Cell wall sugar was determined by one dimensional TLC on cellulose plate (Merk, Burlington, MA, USA) using solvent system described by Becker et al. [44] and by LC/MS (AB Sciex TripleTOF 5600+ System) with a CAPCELL CORE C18 (3.0 mm x 100 mm) column using 40% methanol with 0.1% formic acid as the mobile phase after 1-phenyl-3-methyl-5-pyrazolone (PMP) derivatization [42]. The isomer of diaminopimelic acid (DAP) in the cell wall was analyzed using thin-layer chromatography (TLC) as previously described [45]. *N-acyl* group of muramic acid in the peptidoglycan was examined using the previously described method [46]. Mycolic acids were determined using TLC according to the procedure developed by Tomiyasu [47]. Phospholipids in the cell were extracted and determined by two-dimensional TLC following the method of Minikin et al. [48]. Cellular fatty acid methyl esters were extracted and analyzed using the Sherlock microbial identification system (version 6.1; MIDI database: TSBA6, Newark, DE, USA) according to procedure of Sasser [49] by the Scientific Instrument Center, King Mongkut’s Institute of Technology Ladkrabang (KMITL), Thailand. Menaquinones were extracted and purified, using a previously described method [50], and analysed by LC-MS (JSM-T100LP; JEOL, Tokyo, Japan) with a Capcell pak C18 UG 120 column (Shiseido) using methanol:iso-propanol (7:3, v/v) as the mobile phase.

### 2.6. Genome Sequencing and Analysis

Genomic DNA of strain GKU 173^T^ and *F. feengrottensis* HKI 0501^T^ were extracted from the culture grown in Bennett’s broth at 28 °C for seven days according to standard procedure [22]. Whole genomes of strain GKU 173^T^ and *F. feengrottensis* HKI 0501^T^ were sequenced using Illumina Hiseq PE 150 serviced by Novogene (Beijing, China) and PacBio Sequel systems at Chulabhorn Royal Academy, Thailand. Prior to assembly, the reads were trimmed for adapter sequences and filtered for sequence quality and checked by Fast QC tool [51]. Sequencing data were assembled with Unicycler [52] and determined by QUAST [53]. G+C content was calculated from the whole genome sequences. 

Average Nucleotide Identity (ANI) and Ortho ANI [54] were compared using ChunLab’s online ANI calculator [55] and the Orthologous Average Nucleotide Identity tool version 0.93.1 (OAT) [50]. Digital DNA-DNA hybridization (dDDH) was performed using Genome-to-Genome Distance Calculator (GGDC) [56]. Contamination Estimator by 16S software (ContEst16S) was used to determine contamination of 16S rRNA gene from the query genome [57]. Functional annotation was carried out using the Rapid Annotation using Subsystem Technology (RAST) server with the seed database [58] and the potential biosynthetic gene clusters were predicted by antiSMASH (version.5.0.0) [59].

## 3. Results and Discussion

### 3.1. Isolation and Plant Growth Promoting (PGP) Traits

**Figure 1:**
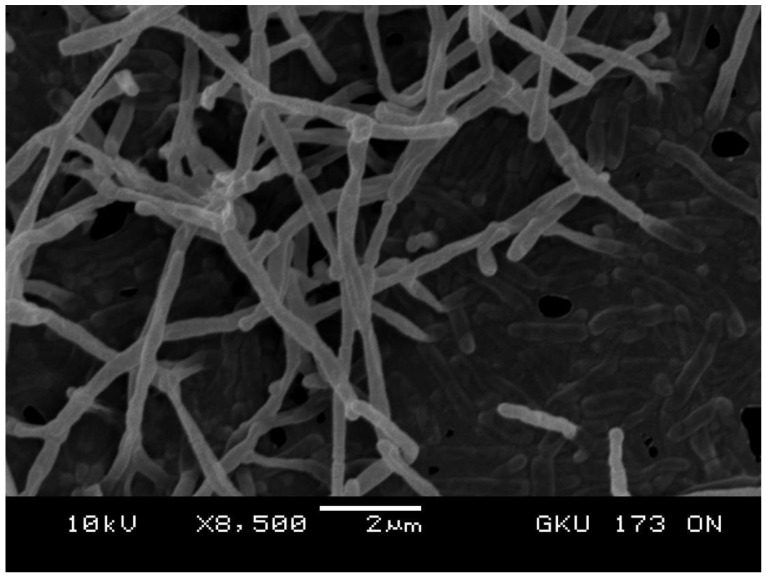
Scanning electron micrograph of fragmentation of aerial hyphae of strain GKU 173^T^ on ON agar at 28 °C after 21 days. Bar, 2 μm.

Strain GKU 173^T^ was isolated from surface-sterilized roots of the black wattle tree (*Acacia mangium* Willd.) on starch-casein agar. It is an aerobic Gram-stain-positive non-sporulating actinobacterium. Morphological observation on ON agar indicated that strain GKU 173^T^ formed a branched substrate mycelium and abundant white aerial hyphae that fragmented into irregular rod-like elements (Figure 1). Strain GKU 173^T^ showed good to abundant growth on all tested media. The substrate mycelium was brownish red, greyish red, high red, and deep red to violet brown associated with red soluble pigment on ISP 2, ISP 3, organic medium 79, Bennett’s agar, and MS agar (Table S1). 

Strain GKU 173^T^ produced IAA at low concentration at an average of 3 ± 0.3 μg mL ^− 1^. It produced a clear and visible dissolution halo on Pikovskaya’s medium supplemented with 2% (w/v) tricalcium phosphate (Figure S1a) indicating its phosphate solubilization activity. It produced siderophores by showing a yellow halo surrounding the bacterial plug on blue agar CAS assay (Figure S1b). The strain was unable to grow on minimum medium supplemented with 3 mM ACC as a nitrogen source, implying no ACC deaminase activity (data not shown). By carrying these PGP-traits, strain GKU 173^T^ might be able to promote plant growth and play a beneficial role to the plants similar to other endophytic actinomycetes [3,4,5].

### 3.2. Phylogenetic Analysis of 16S rRNA Gene

**Figure 2:**
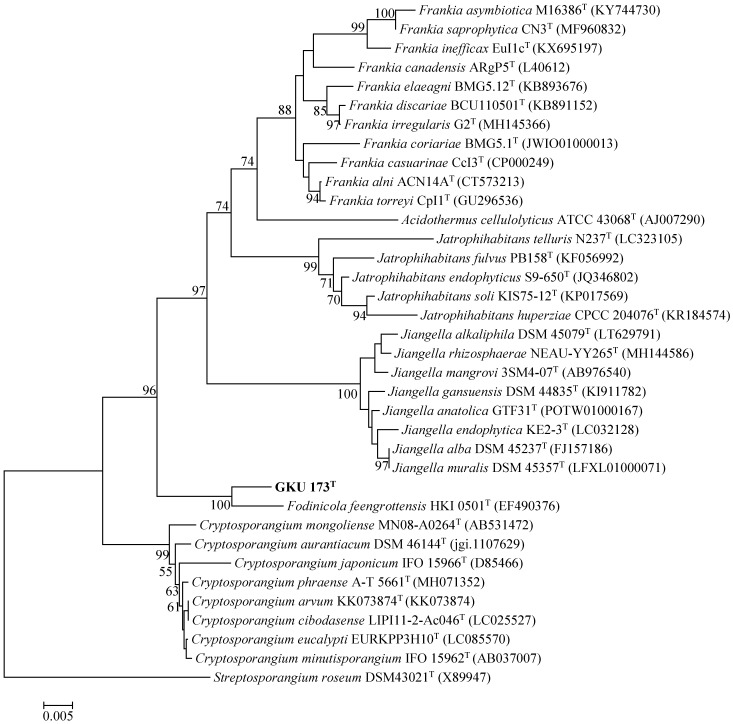
Neighbor-joining phylogenetic tree on the basis of 16S rRNA gene sequences of strain GKU 173^T^ and related species. *Streptosporangium roseum* DSM43021^T^ was used as an outgroup. Numbers at nodes refer to bootstrap values (based on 1000 replicates; only values >50% are shown). Bar, 0.005 nucleotide substitutions per site.

Results of 16S rRNA gene sequence analysis (1498 bp, GenBank accession number MK323078) indicated that strain GKU 173^T^ was the most closely related to the genus *Fodinicola*. The highest similarity values were detected with the only species in the genus, *Fodinicola feengrottensis* HKI 0501^T^ (97.13%). The strain also showed a remote relationship (<95% similarity) with other species including *Cryptosporangium eucalypti* EURKPP3H10^T^ (94.64%), *Cryptosporangium phraense* A-T 5661^T^ (94.57%), *Cryptosporangium aurantiacium* DSM 46144^T^ (94.50%), *Jiangella anatolica* GTF31^T^ (94.36%), *Cryptosporangium minutisporangium* IFO 15962^T^ (94.36%), and *Frankia coriariae* BMG5.1^T^ (94.34%). Neighbor-joining phylogenetic analysis indicated that strain GKU 173^T^ grouped tightly with the only member of the genus *Fodinicola* (Figure 2). The positions of strain GKU 173^T^ in all reconstruction trees were in agreement and supported by the high bootstrap values (Figures S2 and S3). Genus *Fodinicola* proposed by Carlsohn et al. [11] is monophyletic and forms a distinct cluster with the genus *Cryptosporangium* [14] as a member of the family *Cryptosporangiaceae* [12] within the order *Cryptosporangiales* [13]. At the time of writing, this genus comprises only a single species, *F. feengrottensis* HKI 0501^T^, which was isolated from acidic rocks of a medieval alum slate mine in Germany in 2008 [11]. Based on phylogenetic analysis, strain GKU 173^T^ is likely to belong to the genus *Fodinicola.*

### 3.3. Phenotypic Characterization

Strain GKU 173^T^ and its closely related species, *F. feengrottensis* HKI 0501^T^, were phenotypically characterized. Strain GKU 173^T^ showed better growth than *F. feengrottensis* HKI 0501^T^ in most tested media (Table S1). Its substrate mycelium was in range of red associated with red soluble pigment, while mycelium of strain HKI 0501^T^ was pale orange with no soluble pigment (Table S1). Strain GKU 173^T^ was able to grow between 14 to 42 °C, with optimal growth at 24 to 30 °C. The pH range for growth was 4–11, with optimal growth at pH 7 and 8. The strain grew in NaCl concentrations of 0%–6% (w/v) with good growth at concentration up to 3% (w/v). Strain GKU 173^T^ showed oxidase-negative and catalase-positive. The difference of acidic production, and carbon/nitrogen utilization of strain GKU 173^T^ and *F. feengrottensis* HKI 0501^T^ are summarized in Table 1. Based on morphological and phenotypical data, strain GKU 173^T^ can be distinguished from *F. feengrottensis* HKI 0501^T^.

### 3.4. Chemotaxonomic Analysis 

The analysis of the cell wall peptidoglycan by TLC (Figure S4) and LC/MS showed that strain GKU 173^T^ contained meso-diaminopimelic acid, D-alanin, D-glutamic acid, and glycine. Cell wall sugars were xylose and mannose. The *N*-acyl type of muramic acid in the peptidoglycan was N-acetyl. Mycolic acids were absent. The polar phospholipids comprised of diphosphatidylglycerol (DPG), phosphatidylethanolamine (PE), phosphatidylinositol (PI), several unknown phospholipids, and unknown ninhydrin-positive compounds (Figures S5 and S6). The fatty acid profiles predominantly contained iso-C_16:0_ (58.22%), anteiso-C_17:0_ (9.94%), iso-C_18:0_ (9.35%), and iso-C_17:0_ (6.58%); and a small proportion of 10-methyl C_17:0_ (3.87%), 10-methyl C_18:0_ (1.9%), iso-C_16:1_ (1.63%), sum in feature 8 (C_18:1_ *ω*6c, 1.3%), and summed feature 8 (C_18:1_ *ω*7c or C_18:1_ *ω*6c, 1.3%). The menaquinones were MK-9(H_4_) (25%), MK-9(H_6_) (36%) and MK-9(H_8_) (39%). All chemotaxonomic results revealed that strain GKU 173^T^ shared apparent characteristics with the only member of the genus *Fodinicola* particularly, the presence of xylose as the diagnostic cell-wall sugar [11]. Altogether, chemotaxonomic and phenotypic data evidently constitute strain GKU 173^T^ as a novel species within the genus *Fodinicola*.

### 3.5. Whole Genome Sequencing and Digital DNA-DNA Hybridization (dDDH)

Genomes of strain GKU 173^T^ and strain HKI 0501^T^ were sequenced and compared. The assembled draft genomes of strain GKU 173^T^ and strain HKI 0501^T^ contained 4 and 107 contigs with a total length of 8.86 Mbp (GenBank accession number WOTN00000000) and 8.71 Mbp (GenBank accession number WOTO00000000), respectively. The N50 value of genomes of strains GKU 173^T^ and HKI 0501^T^ was 2.35 Mbps (L50 = 2) and 1.15 Mbps (L50 = 26), respectively. The ANI value between strain GKU 173^T^ and strain HKI 0501^T^ was 77.92% which was much lower than the ANI threshold range (95%–96%) for species delineation [60]. The dDDH value between both strains was 21.30%, which was much lower than the recommended cut-off value of 70% for species recognition [61]. The calculated genomic G+C contents of strain GKU 173^T^ and *F. feengrottensis* HKI 0501^T^ were 67.81% and 66.61%, respectively. The difference in G+C value was higher than 1% indicating distinct species according to Meier-Kolthoff et al. [62]. On the basis of polyphasic characteristics, strain GKU 173^T^ was distinguished from the closely related type strain and represents a novel species of the genus *Fodinicola*, for which the name *Fodinicola acaciae* sp. nov. (= TBRC 10620^T^ = NBRC 114213^T^) is proposed.

### 3.6. Description of Fodinicola acaciae sp. nov.

*Fodinicola acaciae* (a.ca.ci’ae. L. n. acacia, the acacia-tree and also the name of a botanical genus; L. gen. n. *acaciae*, of *Acacia*, isolated from roots of a black wattle tree (*Acacia mangium* Willd.)

Gram-stain-positive, aerobic, non-motile, catalase-positive, oxidase-negative actinomycete that produces branched substrate mycelium and abundant aerial mycelium. Aerial hyphae break up into irregular rod-like elements. Colonies are wrinkled, beige to orange in color. Red-series diffusible pigment is produced on Bennett, ISP 2, ISP 3, organic medium 79, and MS media. Growth occurs between 14 and 42 °C, good growth at 28 °C, and no growth below 14 °C or above 42 °C. Good growth occurs at pH 7.0 and pH 8.0, but no growth occurs at pH 3 nor pH 12. NaCl tolerance is up to 6% (w/v). Nitrate is not reduced. Gelatin liquefaction, milk coagulation and peptonization are positive. Acid production from adonitol, L-arabinose, D-fructose, glycerol, D-glucose, *myo*-inositol, lactose, mannitol, D-raffinose, D-sorbitol, sucrose, D-tretalose, and D-xylose, but not from rhamnose. L-tyrosine, Tween 20, and Tween 80 are degraded, but adenine, hypoxanthine, starch, urea, xanthine, and xylan are not. Adonitol, L-arabinose, D-fructose, D-glucose, glycerol, *myo*-inositol, lactose (weakly), mannitol, D-raffinose, rhamnose, D-sorbitol, sucrose, D-trehalose, and D-xylose are used as carbon sources. L-arginine, L-cysteine, L-histidine, L-isoleucine, L-phenylalanine, potassium nitrate, L-tryptophan, and L-valine are used as nitrogen sources. By API ZYM system, production of acid phosphatase, alkaline phosphatase, α-chymotrypsin (weakly), cysteine arylamidase (weakly), esterase (C4), α-galactosidase, β-galactosidase, N-acetyl-β-glucosaminidase, α-glucosidase, β-glucosidase, leucine arylamidase, lipase (C8), lipase (C14), α-mannosidase, naphthol-AS-BI-phosphohydrolase, and trypsine are positive, but not α-fucosidase and β-glucuronidase. The cell wall peptidoglycan contains meso-A_2_pm, D-alanine, D-glutamic acid, and glycine. The muramic acid in peptidoglycan is *N*-acetylated. The cell-wall sugars are xylose and mannose. The predominant menaquinones are MK-9(H4), MK-9(H6), and MK-9(H8). The polar phospholipids comprise diphosphatidylglycerol (DPG), phosphatidylethanolamine (PE), phosphatidylinositol (PI), several unknown phospholipids, and unknown ninhydrine-positive compounds. Mycolic acids are absent. The predominant cellular fatty acid profiles contain iso-C_16:0_, anteiso-C_17:0_, iso-C_18:0_, and iso-C_17:0_. The G+C content is 67.81% (determined from the genome sequence).

Type strain GKU 173^T^ (= TBRC 10620^T^ = NBRC 114213^T^) was isolated from the roots of black wattle tree (*Acacia mangium* Willd.) collected at Kasetsart University, Bangkok, Thailand. 

### 3.7. PGP-Trait and Biosynthetic Gene Cluster (BGC) Prediction

RAST annotation of genome sequences of strain GKU 173^T^ showed 8705 coding sequences and 55 RNAs, while *F. feengrottensis* HKI 0501^T^ showed 11,978 coding sequences and 57 RNAs. The genome annotation revealed genes related to the PGP traits according to those in vitro activities of strain GKU 173^T^ and HKI 0501^T^ (Table 2). Indole-3-acetamide (IAM) hydrolase representing the key enzyme converting IAM to IAA in IAA biosynthetic pathway [63] was located in the genome of strain GKU 173^T^, while absent in that of strain HKI 0501^T^. Both genomes consisted of two to three copies of acid phosphatase genes that are likely involved with inorganic phosphate solubility [64]. The genome of strain GKU 173^T^ exhibited siderophore biosynthetic gene cluster (BGC) of lucA/lucC family protein [65].

The BGCs from genomes of strain GKU 173^T^ and *F. feengrottensis* HKI 0501^T^ were investigated by antiSMASH. The results showed that strain GKU 173^T^ carried 16 BGCs including 5 known BGCs and 11 uncharacterized BGCs (Table 2). The silent/cryptic BGCs encoded 6 ribosomally synthesized and post-translationally modified peptides (RiPPs), 1 siderophore, 1 Type I polyketide (PK)-non-ribosomal peptide (NRP) hybrid, 1 Type II PK, and 2 terpenes. The genome of strain HKI 0501^T^ consisted of 16 BGCs including 2 known BGCs and 14 uncharacterized BGCs (Table 2). The silent/cryptic BGCs encoded 3 NRPs, 2 RiPPs, 1 siderophore, 2 Type I PKs, 2 Type II PKs, 1 Type I PK-NRP hybrid, and 3 terpenes. Interestingly, the Type I PK-NRP BGCs of both strains were nearly identical (98%) and organized in the same pattern, thus, they might be involved in the same biosynthesis of new compounds. The in-silico genome analysis above supported that rare actinomycetes carried significant number of BGCs, particularly the silent/cryptic clusters [10,66]. Therefore, members of genus *Fodinicola* are potential species for production of novel competent compounds. 

## 4. Conclusions

Based on the polyphasic taxonomic study demonstrated that strain GKU 173^T^ isolated from roots of a black wattle tree (*Acacia mangium* Willd.) constitutes a novel species within the genus *Fodinicola* of which *Fodinicola acaciae* sp. nov. was proposed under the type strain GKU 173^T^ (= TBRC 10620^T^ = NBRC 114213^T^). Genome analysis of the novel strain and the closely related strain, *F. feengrottensis* HKI 0501^T^, revealed potential PGP-traits including genes involved in phosphate solubilization, IAA and siderophore production; and a variety of uncharacterized BGCs comprising NRPs, RiPPs, Type I PKs, Type II PKs, Type I PK-NRP hybrid, and terpenes. The results suggested that members in the genus *Fodinicola* have potential activity as beneficial PGP-bacteria and specialized secondary metabolite producers. 

## Figures and Tables

**Table 1:** Differential characteristics of strain GKU 173^T^ and closely related *Fodinicola* species. All data obtained from the present study unless otherwise indicated. +, Positive; w, weakly positive; -, negative.

Characteristics	GKU 173^T^	*Fodinicola feengrottensis* HKI 0501^T^
Isolation source	Roots of plants	Acidic rocks *
Cell morphology	Fragmentation of aerial hyphae	Fragmentation of aerial hyphae *
Growth Substrate mycelium Soluble pigment	Good Red Red	Moderate Pale orange None
Temperature range for growth (°C)	14–42	14–40
pH range for growth	4–11	4–9
NaCl range for growth (%, w/v)	0–6	0–3
Urea	-	+
Acid production:		
- Adonitol	+	-
- Myo-inositol	+	-
- Rhamnose	-	+
- D-sorbitol	+	-
Carbon utilization:		
- Adonitol	+	-
- Lactose	w	+
- Myo-inositol	+	-
- Rhamnose	+	w
- D-sorbitol	+	-
- D-xylose	+	w
Nitrogen utilization:		
- L-histidine	+	w
- L-isoleucine	+	w
- L-tryptophan	+	-
- L-valine	+	w
Enzyme activities:		
- Βeta-glucosidase	+	-
- Cystine arylamidase	w	+
- Esterase	+	w
- Lipase	+	w

* Data obtained from [11].

**Table 2:** Genome analysis of PGP-trait and biosynthetic gene cluster (BGC) prediction of strain GKU173^T^ and *Fodinicola feengrottensis* HKI 0501^T.^

Gene Feature	GKU 173^T^	HKI 0501^T^
**PGP-trait Prediction**		
- IAA production	Indole-3-acetamide (IAM) hydrolase	-
- Phosphate solubilization	Acid phosphatases	Acid phosphatases
- Siderophore production	- IucA/IucC family siderophore biosynthesis proteins - N-acetyltransferase - Aminotransferase	- IucA/IucC family siderophore biosynthesis proteins - N-acetyltransferase - Aminotransferase
**BGC Prediction** (**number**)		
**Known BGCs**		
- Bacteriocin	1	1
- Ectoine	1	1
- Geosmin	1	0
- Hopene	1	0
- 2-methylisoborneol	1	0
**Silent/cryptic BGCs**		
- Lanthipeptide	5	2
- Lassopeptide	1	0
- Non-ribosomal peptide (NRP)	0	3
- Siderophore	1	1
- Type I polyketide (PK)	0	2
- Type I PK-NRP hybrid	1	1
- Type II PK	1	2
- Terpene	2	3

## Supplementary Materials

The following are available online at https://www.mdpi.com/2076-2607/8/4/467/s1.
