# Supplementary material for: Fodinicola acaciae sp. nov., an Endophytic Actinomycete Isolated from the Roots of Acacia mangium Willd. and Its Genome Analysis
Source: Microorganisms. 2020 Mar 25;8(4):467. doi: 10.3390/microorganisms8040467 (PMC7232338; doi:10.3390/microorganisms8040467)
Supplement: Supplementary file 1 [file microorganisms-08-00467-s001.pdf]

Fodinicola acaciae sp. nov., an endophytic actinomycete isolated from the roots of _Acacia mangium_ Willd. and its genome analysis

Huyền Thị Thanh Phạm[1,2], Wipawadee Suwannapan[1], Wilaiwan Koomsiri[1,2], Yuki Inahashi,[3] Akira Také,[4] Atsuko Matsumoto,[3] Arinthip Thamchaipenet[1,2]*

[1]Department of Genetics, Faculty of Science, Kasetsart University, Chatuchak, Bangkok 10900, Thailand

[2]Omics Center for Agriculture, Bioresources, Food and Health, Kasetsart University (OmiKU), Bangkok 10900, Thailand

[3]Kitasato Institute for Life Sciences, Kitasato University, 5-9-1 Shirokane, Minato-ku, Tokyo 108-8641, Japan

[4]Department of Microbiology, Kitasato University School of Medicine, 1-5-1 Kitasato, Minami-ku, Sagamihara, Kanagawa 252-0374, Japan

*Author for correspondence:

Arinthip Thamchaipenet

Tel: +66-2-562-5444 ext. 646703; Fax: +66-2-579-5528; E-mail: arinthip.t@ku.ac.th

Table S1. Cultural characteristics of strain GKU 173^T and *Fodinicola feengrottensis* HKI 0501^T at 28 ^{o}C for 21 days

Media	Characteristics	GKU 173^T	HKI 0501^T
ISP 2	Growth	Abundant	Poor
	Aerial mycelium	Greyish red, white	Orange
	Substrate mycelium	Wrinkled, brownish red	Orange
	Soluble pigment	Deep red	None
ISP 3	Growth	Abundant	Good
	Aerial mycelium	White	White, pale orange
	Substrate mycelium	Pastel red	Wrinkled, orange
	Soluble pigment	Greyish red	None
ISP 4	Growth	Abundant, flat	Moderate
	Aerial mycelium	White	Pale orange
	Substrate mycelium	Light orange	Wrinkled, pale orange
	Soluble pigment	None (clear zone)	None
ISP 5	Growth	Abundant, flat	Poor
	Aerial mycelium	White	Sparse white, orange
	Substrate mycelium	Pale orange to pastel red	Pale orange
	Soluble pigment	None	none
ISP 6	Growth	Good	Moderate
	Aerial mycelium	Creamy, pale orange	Creamy, light yellow
	Substrate mycelium	Wrinkled, pale orange	Wrinkled, light orange
	Soluble pigment	None	None
ISP 7	Growth	Abundant, flat	Poor
	Aerial mycelium	White	Orange
	Substrate mycelium	Light orange to pale orange	Pale orange
	Soluble pigment	None	None
Bennett's agar	Growth	Abundant	Abundant
	Aerial mycelium	White with brownish gray spots	Pale orange
	Substrate mycelium	Wrinkled, deep red	Wrinkled, pale orange
	Soluble pigment	Deep red	None
Organic medium 79	Growth	Abundant	Good
	Aerial mycelium	Short white, pastel red	Pale orange, creamy
	Substrate mycelium	Wrinkled, orange-red to high red	Wrinkled, pale orange
	Soluble pigment	High red	None
MS	Growth	Abundant	Abundant
	Aerial mycelium	White	Pale orange
	Substrate mycelium	Violet Brown	Pale orange
	Soluble pigment	Violet Brown	None

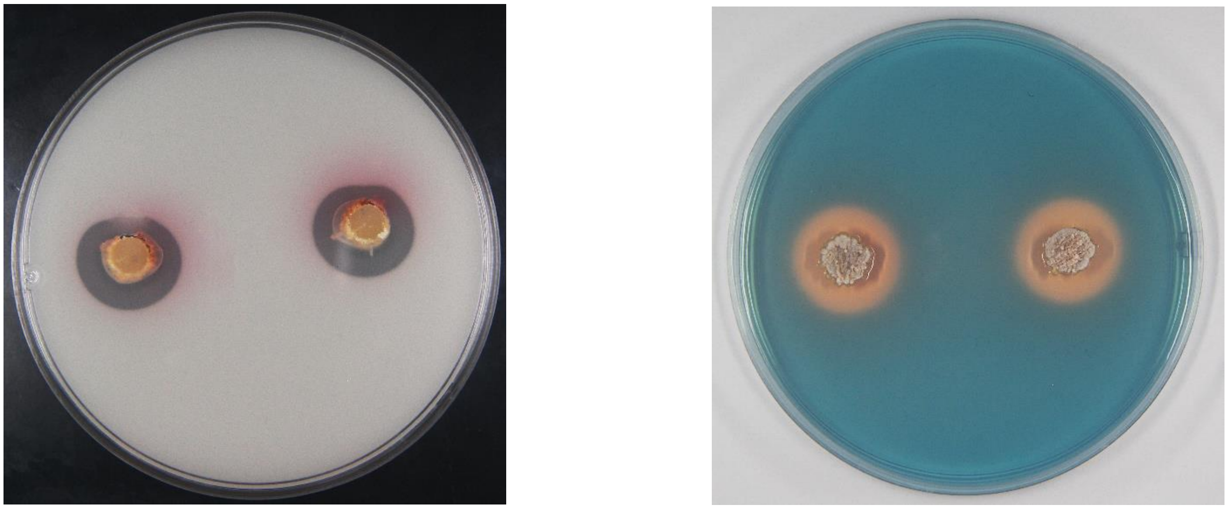

(a) **(b)**

Figure S1. PGP-traits of strain GKU 173^{T}. (a) Phosphate solubilization on Pikowskaya's agar supplemented with 2 % tricalcium phosphate. (b) Siderophore production on CAS agar.

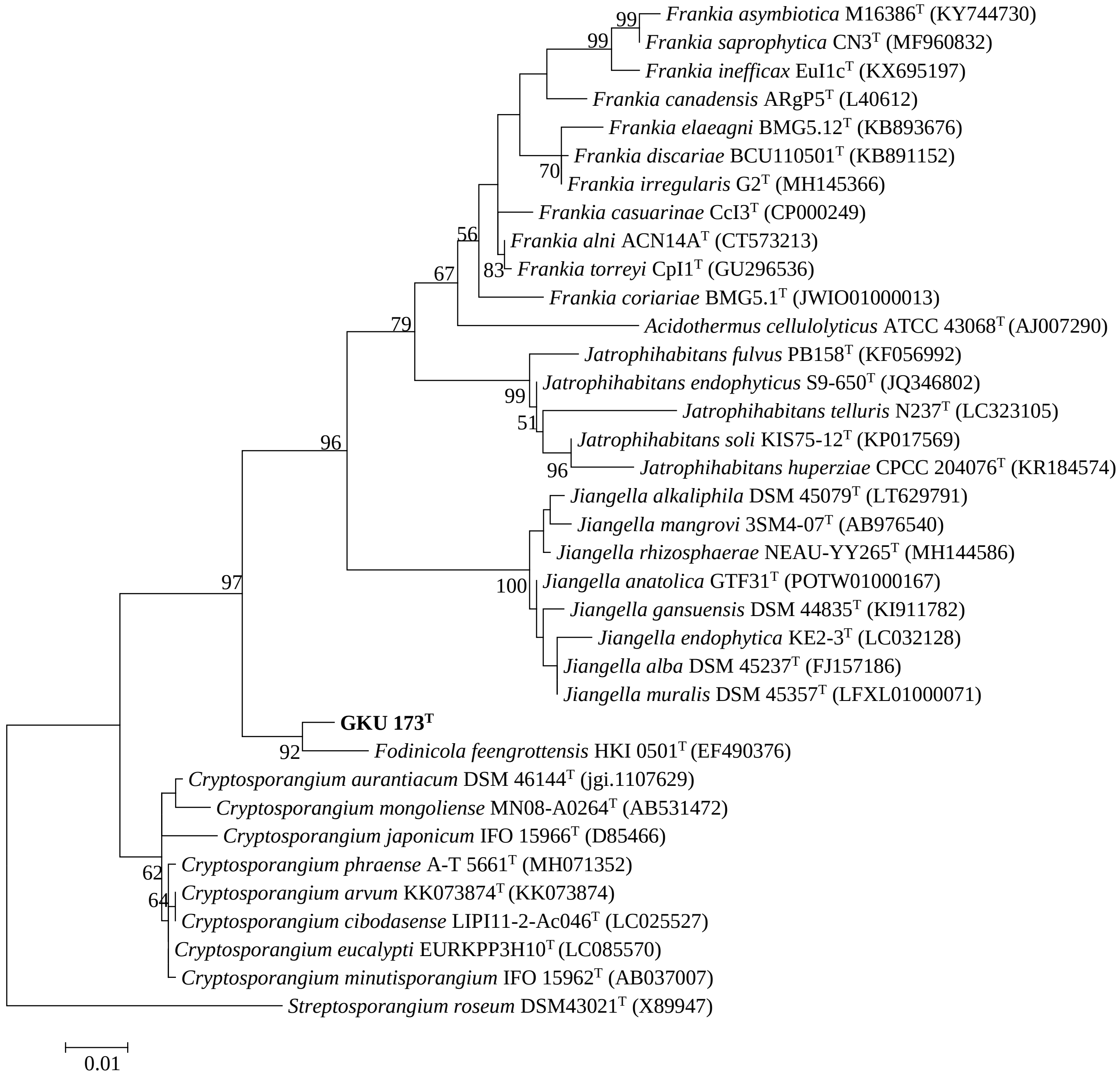

Figure S2. Maximum-likelihood phylogenetic tree on the basis of 16S rRNA gene sequences of strain GKU 173^{T} and related species. *Streptosporangium roseum* DSM43021^{T} was used as an outgroup. Numbers at nodes refer to bootstrap values (based on 1000 replicates; only values >50 % are shown). Bar, 0.01 nucleotide substitutions per site.

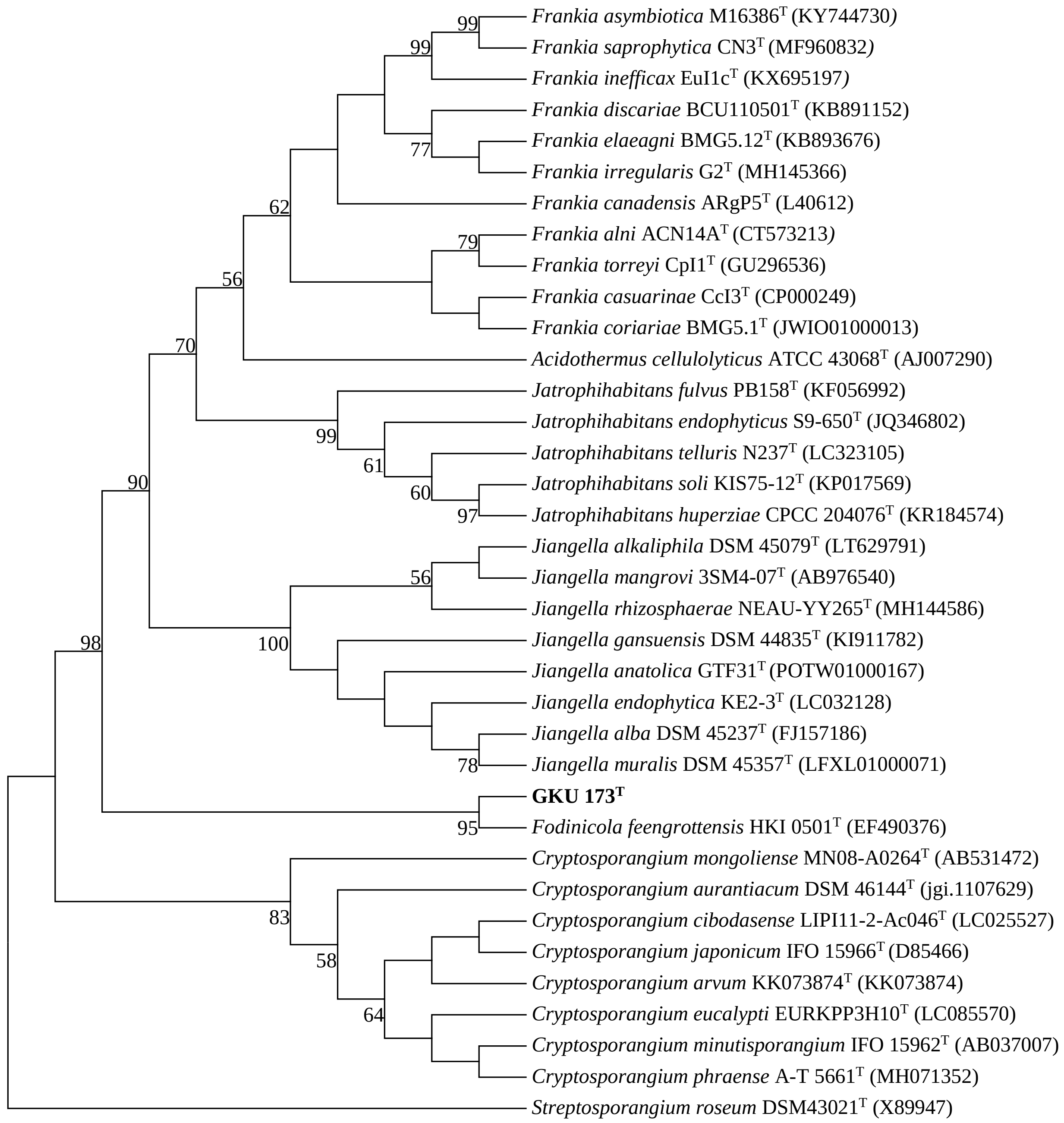

Figure S3. Maximum-parsimony phylogenetic tree on the basis of 16S rRNA gene sequences of strain GKU 173^{T} and related species. *Streptosporangium roseum* DSM43021^{T} was used as an outgroup. Numbers at nodes refer to bootstrap values (based on 1000 replicates; only values >50 % are shown).

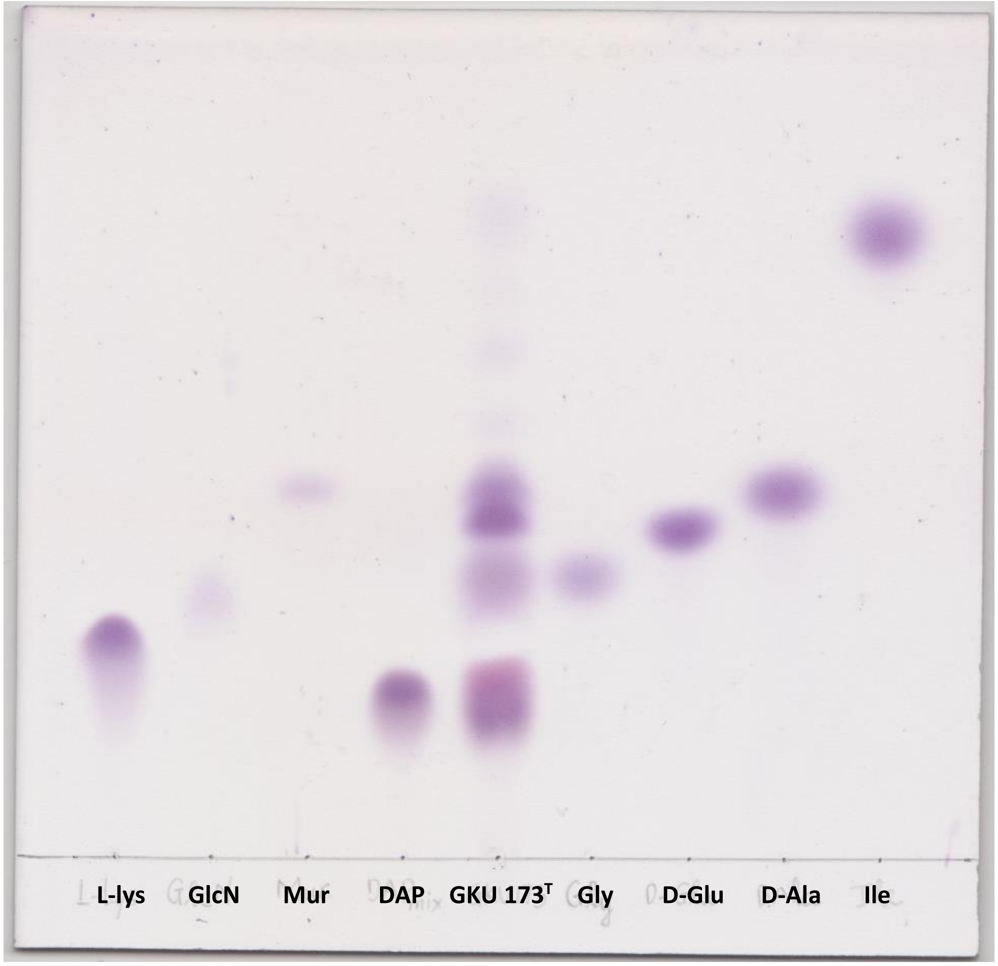

Figure S4. Analysis of the cell wall peptidoglycan by one dimensional TLC on cellulose plate. Abbreviation: D-ala, D-alanine, DAP, diaminopimelic acid, GlcN, glucosamine, D-glu, D-glutamic acid, Gly, glycine, Ile, isoleucine, L-lys, L-lysine, Mur, muramic acid

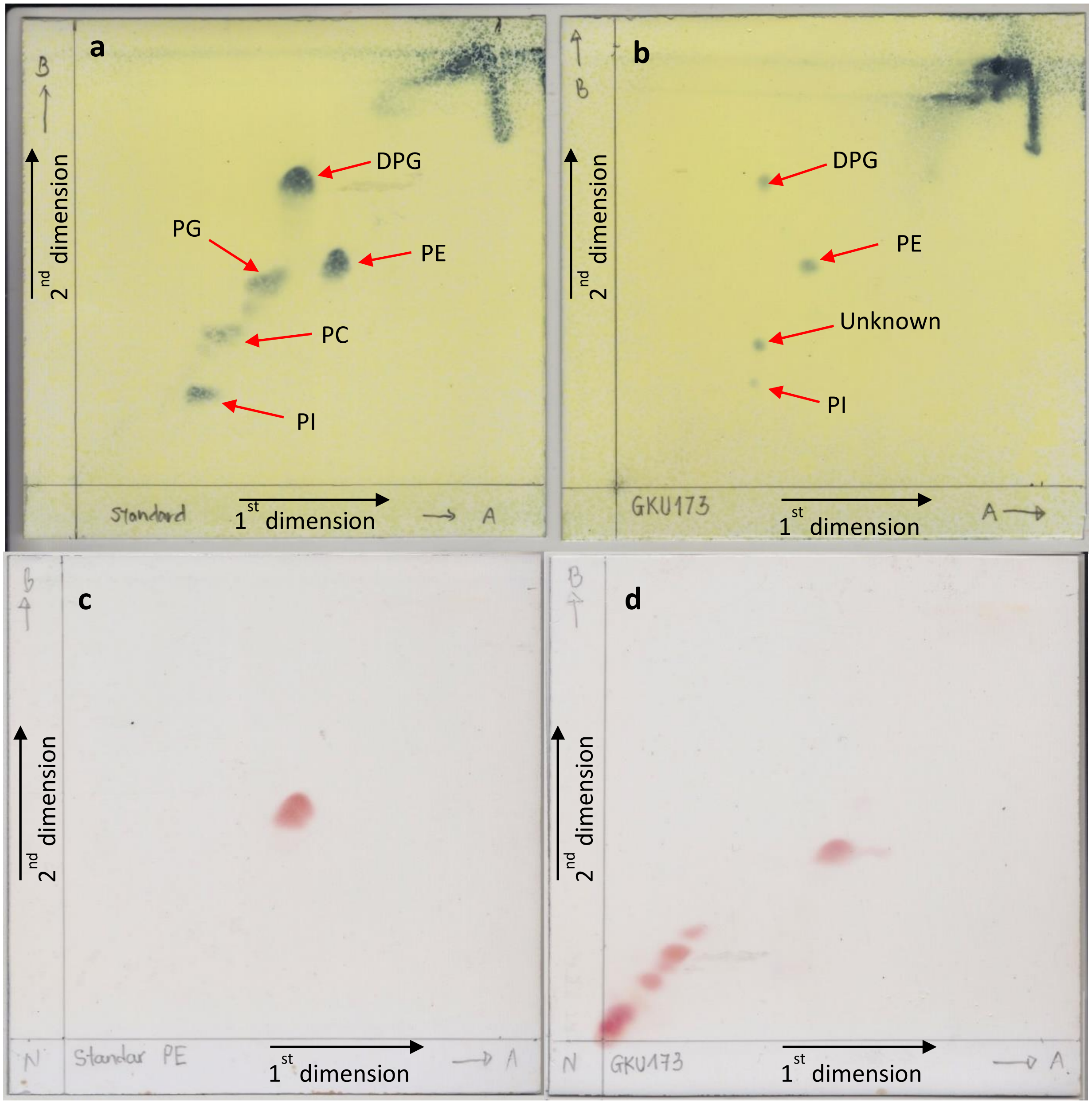

Figure S5. Phospholipids analysis separated by two-dimensional TLC. First and second dimension conditions are chloroform:methanol:water (65:25:4) and chloroform:acetic acid:methanol:water (40:7.5:6:2), respectively. (a) Phospholipid standard sprayed with phosphomolybdic acid reagent, (b) phospholipids of strain GKU 173^T sprayed with phosphomolybdic acid reagent, (c) PE standard sprayed with ninhydrin reagent, (d) phospholipids of strain GKU 173^T sprayed with ninhydrin reagent. Abbreviation: DPG, diphosphatidylglycerol; PC, phosphatidylcholine; PE, phosphatidylethanolamine; PG, phosphatidylglycerol; PI, phosphatidylinositol

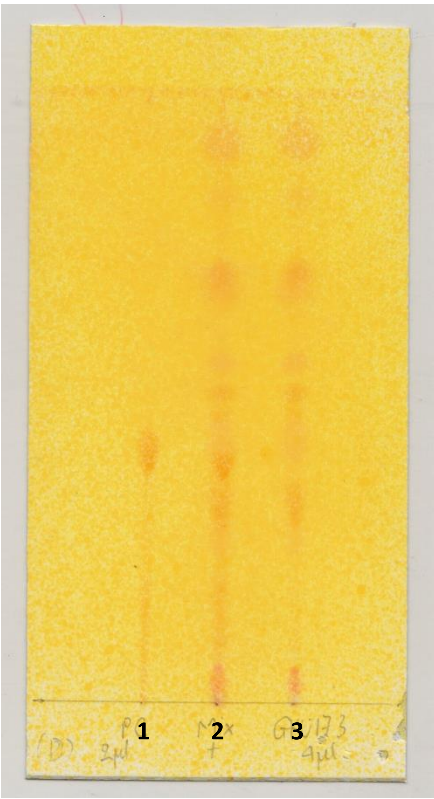

Figure S6. Phospholipids analysis separated by one-dimensional TLC and sprayed with dragendorff reagent. Lane 1, phosphatidylcholine (PC) standard; Lane 2, mixture of PC and phospholipids of strain GKU 173^{T}; Lane 3, phospholipids of strain GKU 173^{T}.
